# Repetition Blindness for Natural Images of Objects with Viewpoint Changes

**DOI:** 10.3389/fpsyg.2012.00622

**Published:** 2013-01-22

**Authors:** Stéphane Buffat, Justin Plantier, Corinne Roumes, Jean Lorenceau

**Affiliations:** ^1^Institut de Recherche Biomédicale des ArméesBrétigny sur Orge, France; ^2^Université Pierre et Marie Curie-Paris 6, Centre de Recherche de l’Institut du Cerveau et de la Moelle épinière, UMR S975Paris, France; ^3^INSERM, U975Paris, France; ^4^CNRS, UMR 7225Paris, France

**Keywords:** object recognition, natural images, repetition blindness, spatial frequency, viewpoint dependence

## Abstract

When stimuli are repeated in a rapid serial visual presentation (RSVP), observers sometimes fail to report the second occurrence of a target. This phenomenon is referred to as “repetition blindness” (RB). We report an RSVP experiment with photographs in which we manipulated object viewpoints between the first and second occurrences of a target (0°, 45°, or 90° changes), and spatial frequency (SF) content. Natural images were spatially filtered to produce low, medium, or high SF stimuli. RB was observed for all filtering conditions. Surprisingly, for full-spectrum (FS) images, RB increased significantly as the viewpoint reached 90°. For filtered images, a similar pattern of results was found for all conditions except for medium SF stimuli. These findings suggest that object recognition in RSVP are subtended by viewpoint-specific representations for all spatial frequencies except medium ones.

## Introduction

Successful interaction with the visual world depends on the ability to recognize visual objects quickly and accurately, despite countless variations in their appearance and settings. However, understanding how the brain can reliably build an object representation in conditions of ever-changing retinal stimulation remains a challenge. Especially, experiments that concentrated on the effect of rotation in depth on the recognition of tridimensional objects provided substantial evidence in favor of multiple visual recognition systems (Logothetis and Sheinberg, 1996; Tarr, 2004).

A useful framework to investigate object recognition is based on a multi-channel processing corresponding to different spatial frequency (SF) bands. The parvocellular visual pathway, which is predominantly involved in the processing of high SFs (HSFs), has slower responses than the magnocellular visual pathway, which processes low SFs (LSFs; Legge, 1978). These observations, which suggest that the human visual system processes information at different spatial scales separately, have inspired a “frame and fill” model of object recognition (Park et al., 1996; Mc Sorley and Findlay, 2002; Bar, 2003; Calderone et al., 2012). LSFs convey global information about shape, general orientation, and proportion (Bar, 2003), or relationships between object parts (Gosselin, 2001; Bar, 2003; Goffaux et al., 2003, 2005). This global information is used to form proto-object representations, which are compatible with multiple candidate objects. The more fine-grain information provided by HSFs helps to select the correct object among these candidates (Braje et al., 1995). However, the fact that the visual system would use specific SF bands to extract different type of information is challenged by a more flexible use of spatial frequencies. The visual system could instead use several SF bands, either depending on the task (Schyns and Oliva, 1997, 1999), or on the size of an object (Gold et al., 1999) to establish a mapping between distance-dependent absolute SF information and diagnostic object-based SF information.

In this study, we use a repetition blindness (RB) paradigm (Kanwisher, 1987) to investigate viewpoint tolerance with natural objects presented from different viewpoints and defined by different SF contents. We detail this paradigm thereafter, before describing the specific hypotheses of the present study.

Repetition blindness refers to a reduced ability to report the occurrence of a repeated item in a rapid serial visual presentation (RSVP) paradigm. It is observed when the repetition occurs within a 500-ms interval, and when repeated items are identical, or similar. RB has been found for letters, colors, words, and pictures (Kanwisher et al., 1999). The most widely accepted explanation for RB is the “type-token” hypothesis (Kanwisher, 1987; Chun, 1997). When viewed, an object activates an object representation (*type*). The occurrence of this activation is recorded (*token*). During an RSVP stream, instantiations of repeated item (same or overlapping *type*) are often merged. This means that instead of perceiving two occurrences of the same (repeated) object, the observer reports seeing the object only once. Because the representation elicited by the first occurrence was still active, the observer’s visual system could not process the second occurrence as a new one. This interpretation was supported by the results of a signal-detection analysis, which showed decreased sensitivity for the repeated item (Kanwisher et al., 1996). One of the key points is the nature of the identity or similarity in type between the repeated objects. It has been suggested that semantic processing plays an important role in RB, with picture and words depicting the same object (Bavelier, 1994). However, RB has also been observed for pseudo-objects (Arnell and Jolicoeur, 1997), and differences in RB between upright and upside-down objects have been observed (Harris and Dux, 2005); these findings are consistent with a key role of low-level information. This would mean at least part of the RB mechanism could occur at the perceptual level, or be induced by low-level processing. Therefore, a manipulation of the SF content of visual stimuli provides a way for experimenters to investigate the perceptual dimensions of RB. Moreover, RB provides experimenters with an interesting tool to measure perceptual similarity between repeated natural objects.

Using this RB paradigm, we aim at testing the hypotheses that:

(i) Object recognition rates should depend on the SF content of the stimulus, given the size of the objects; (ii) because objects have to be typed and tokenized, the size of the RB effect should also depend on SF as it modulates recognition (Schyns and Oliva, 1997, 1999); (iii) in keeping with the previous study of Hayward et al. (2010), RB should depend on viewpoint differences. Indeed, Hayward et al. (2010) report an increased RB for viewpoint differences between 60° and 90° for gray-scale images but not for line drawings. Because both types of stimuli contain power in each of the various SF bands, and line drawings are in essence a selection of diagnostic features, we expect to find a similar pattern of results for full-spectrum (FS) images.

## Materials and Methods

### Observers

Seven volunteers took part in the experiment (Mean age = 23 years). One was an author; the others were naive to the purpose of the study. All participants provided informed consent in writing, and the internal review board of our research institute (Institut de Recherche Biomédicale des Armées) cleared the experiment. All had normal or corrected-to-normal visual acuity and contrast sensitivity.

### Apparatus and stimuli

**Figure 1:**
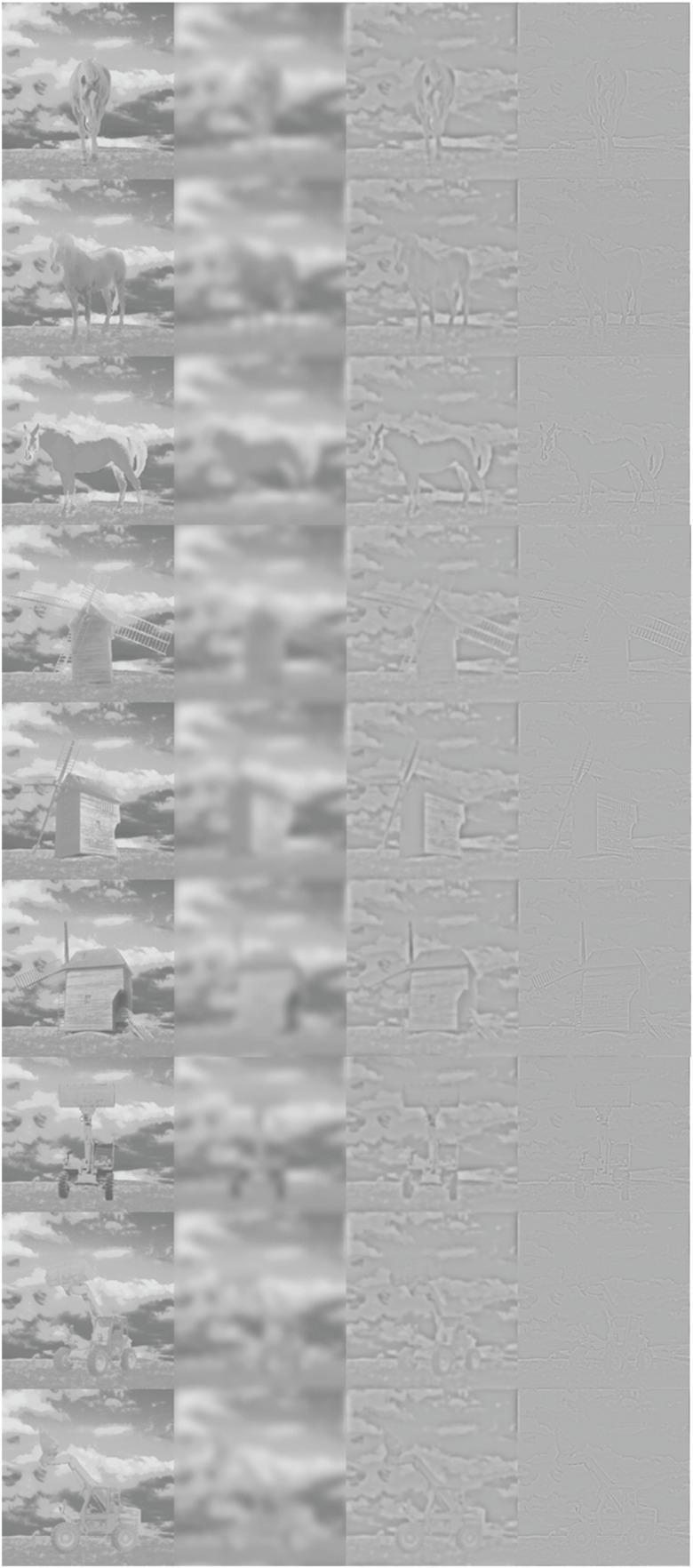
**From left to right: respectively FS, LSF, MSF, and HSF stimuli**. Top to bottom, the three viewpoints for the horse, the windmill, and the backhoe are presented.

Stimuli were displayed on a flat-screen CRT (Iiyama® Vision Master Pro 510), with a diagonal of 22″ and a refresh rate of 200 Hz. The luminance was measured with a Minolta CS100 photometer (mean luminance = 20 cd m^−2^) and the monitor settings were adjusted to produce a gamma function. Observers sat in a dim lit room with their head maintained by a chin-rest. Viewing distance was 120 cm, and the stimuli (512 × 512 pixels) spanned 6.7° × 6.7° of visual angle. Photographs of a horse, a backhoe, and a windmill seen from three different viewpoints (arbitrarily labeled 0°, 45°, and 90°) were transformed into gray-level images to eliminate color cues. These objects were chosen for the following reasons: (i) they have a principal axis and can be decomposed into parts which, in theory, preclude viewpoint-dependency discrepancies (Biederman and Gerhardstein, 1993); (ii) they are typical exemplars of different categories and can be addressed at the same entry level in a semantic network, thus preventing any categorization-bias issue (Jolicoeur et al., 1984). The natural background provided neither semantic nor size or orientation cues. Gray-level images were spatially filtered to generate three types of filtered stimuli with central SFs set at 1.5, 6, and 24 cycles per degree (c/deg), with a width of 1.5 octaves; stimuli filtered in this way are hereafter referred to as LSF, medium SFs (MSF), and HSF stimuli, respectively. Figure 1 shows the amplitude for all images of objects and all orientations. This yielded a total of nine FS images and 27 filtered images (see Figure 1).

**Figure 2:**
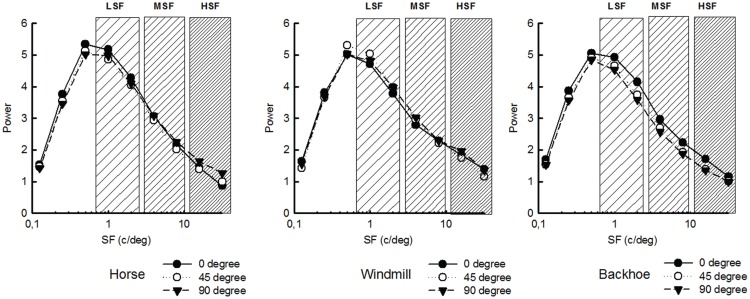
**Amplitude as a function of SF for each image of object at all viewpoints**. Note the high similarity between stimuli. The dashed boxes represent the filters (LSF, MSF, and HSF).

With the filtering method that was used in this study, filtered images have the same energy as the FS image in a given SF band. Since our primary goal was to study the contribution of each SF band, as it exists in natural (FS) images, no inter-band normalization was applied (Costen et al., 1996; see Figure 2).

### Procedure

**Figure 3:**
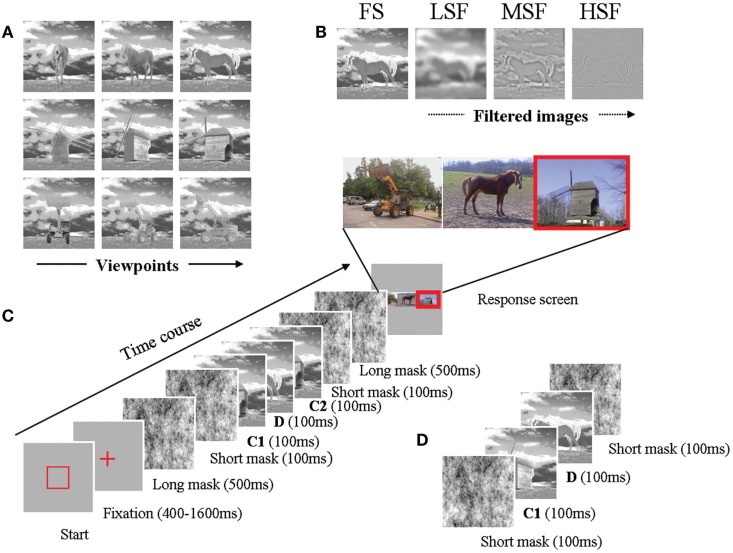
**(A)** All viewpoints for all objects. Note that in our experiment, we consider viewpoint differences between C1 and C2, and not absolute viewpoints. **(B)** Filtered images of a horse, respectively LSF, MSF, and HSF. **(C)** Details a typical repeated trial is exemplified here. When a square was displayed, an input was required from the participant. After the observer had pressed the space bar, a fixation cross appeared on the screen for a variable duration. It was followed by a long mask (500 ms), then by a sequence. The sequence consists in a succession of five pictures presented for 100 ms: the first picture is a mask, followed by the first occurrence of a stimulus (C1), a distractor (D), a second stimulus C2 (second occurrence of the same object type as C1 in repeated condition, different object type in a non-repeated sequence) and another mask. Another long mask followed. The response page was then displayed. **(D)** Illustration of a filler sequence, in which only two objects are displayed.

Subjects triggered the stimulation sequence by pressing a key. A fixation cross appeared in the middle of the screen for 500 (±250) ms, followed by a long mask (500 ms), an RSVP sequence (100 ms per image, no ISI), and a second long mask (see Figure 3). Within a trial, masks were the same wavelet white noise, but were randomly rotated (by 90°, 180°, or 270°) to change the spatial distribution of the contrast. The rest of the screen displayed a constant gray field at the mean stimulus luminance.

Three types of sequences were used in the experiment:

“Repeated” sequences consisted of a succession of mask, critical item 1 (C1), distractor (D), critical item 2 (C2), and mask. C1 and C2 consisted of the same target object (same type), with a possible change in viewpoint; D was the picture of an object different from C1; “Non-repeated” sequences consisted of the same succession (mask, C1, D, C2, mask), but with C1 and C2 corresponding to different objects. Filler (“catch”) sequences consisted of a succession of only four pictures (mask, C1, D, and mask). C1 and D always depicted different objects. Such sequences are commonly used in RSVP studies to prevent subjects from systematically reporting three objects.

The response page displayed color images of the three objects in canonical view, in random order. Observers had to indicate (using the keyboard) how many times they had seen each object (from left to right) during the sequence (0, 1, or 2), regardless of viewpoint. Observers only needed to report the first and third image (respectively C1 and C2) in the sequence. They did not need to report what the distractor (D) was. This response procedure was chosen to minimize phonological access, in accord with Kanwisher et al. (1999). Subjects were warned that some objects could be presented twice, and that some sequences could contain two objects only.

We used the same SF content for C1, D, and C2 in each sequence. Thus, C1, the intervening item D, and C2 (when present) were either all FS images, or all spatially filtered in the same band (LSF, MSF, or HSF). There were 324 repeated or non-repeated sequences. We arbitrarily chose 108 filler sequences (out of 216 in total). Every subject saw all sequences twice. This yielded a total of (324 + 108) × 2 = 864 trials, randomly intermixed across three experimental sessions.

Prior to participating in the main experiment, the observers practiced the task for a short training session, during which all possible pairs of SF images were presented.

### Correct-responses, hits, false alarm rates, and RB

Hits were counted for each critical item accurately reported by the observer in a manner similar to Arnell and Jolicoeur (1997). Reports of an object not presented in a trial, or of more occurrences of an object than actually presented, were counted as false alarms. Thus, a correct-response to a trial corresponds to the observer being credited two hits (C1 and C2 in the non-repeated condition or 2 C1 in the repeated condition) and no false alarm. The chance level in the experiment is the combined probability to be credited two hits and no false alarm.

Hits and false alarms were counted on a trial-per-trial basis, and then averaged to compute overall hit rates (Hi) and false alarm rates (Fa), with a weight of 0.5 for hits and 0.25 for false alarms. These coefficients take into account the structure of the sequences described in the procedure, and the respective probabilities of occurrence of each event. Note that the estimated hit rates were not affected by whether or not observers reported the intervening item/distractor (D).

Repetition blindness refers to a reduced ability to report both occurrences of a repeated item in RSVP. It was measured as the difference between the mean correct-response rate in the non-repeated condition and the mean correct-response rate in the repeated condition.

### Factors and analysis

Filler sequences were not taken into account in the analysis. Three independent variables (factors) were manipulated in 2 × 4 × 3 within-subjects design: (i) the first variable (repetition) was defined by whether the object presented as the first critical item was the same as, or different from, the object presented as the second critical item; (ii) the second variable was the SF (with four levels: FS, LSF, MSF, and HSF); (iii) the third variable corresponded to the viewpoint difference between C1 and C2 (0°, 45°, or 90°).

First, correct-responses were analyzed using a three-way repeated-measure ANOVA to assess perceptual tolerance with respect to viewpoint. Separate repeated-measure ANOVAs were also performed for each SF condition. Second, a signal-detection analysis (see Appendix) was performed. This involved comparing the discrimination index (*A*′) and the decision criterion placement (*B*″) across repeated and non-repeated conditions as a function of viewpoint change and SF content (Snodgrass and Corwin, 1988). While *A*′ can provide confirmation of the RB, *B*″ can give additional insight on how the observers performed the task in repeated and non-repeated conditions. Third, we performed another analysis to compare the expected repeated performance with the actual performance on repeated trials if C1 and C2 were independent. According to Arnell and Jolicoeur (1997), if C1 and C2 were processed independently, we could estimate the expected repeated performance by computing the proportion of trials in which C1 and C2 are correctly reported in non-repeated trial. C1 and C2 proportions are then multiplied together to give the combined proportion correct for both items. This estimated proportion correct for repeated trial has to be compared with the measured one for repeated trials, by means of a *t*-test. The rationale behind this analysis is to attribute the RB affecting C2 to the processing of C1, rather than RB being an effect of C2 position in the RSVP.

## Results

### RB with FS and filtered images of natural objects

**Figure 4:**
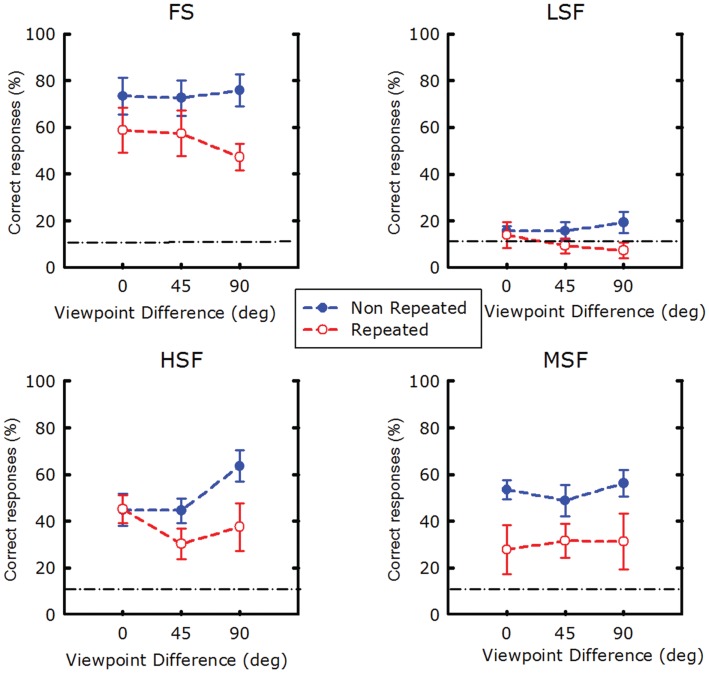
**Correct-responses as a function of viewpoint for each spatial frequency condition**. Data for non-repeated conditions are shown in blue; data for repeated conditions are shown in red. The dark dash-dot line shows the chance level. The chance level in the experiment is the combined probability to be credited two hits and no false alarm. Since the probabilities of a correct-response were equal to 0.33 for C1 and to 0.33 for C2, and assuming that C1 and C2 were recognized independently, the probability of correctly recognizing both C1 and C2 was equal to 0.33 × 2 = 0.11. Error bars show ±1 SEM.

Figure 4 shows the correct-response rates measured in the different test conditions.

#### Main effects

The repeated-measure ANOVA revealed a significant main effect of Repetition [*F*(1, 72) = 82.35, *p* < 0.0001]. Thus, as expected, recognition of a repeated item C2 was worse than recognition of a non-repeated item; this is the RB effect. In addition, there was a main effect of SF [*F*(3, 72) = 47.03, *p* < 0.0001]. Observers usually had high recognition rates for SF, MSF, and HSF stimuli, and markedly lower performance for LSF stimuli.

We found no main effect of viewpoint [*F*(2, 72) = 0.14, *p* = 0.86]. Thus, based on the results of this experiment, viewpoint difference between C1 and C2 seems not an important factor in RB.

#### Interactions

No significant interaction between SF and viewpoint was observed [*F*(2, 72) = 0.36, *p* = 0.905]. This indicates that there was no preferred SF content for a given viewpoint.

However, a significant interaction between repetition and SF was observed [*F*(7, 72) = 3.79, *p* = 0.013], indicating that the SF content of images affects RB. This interaction is also found when we do not take into account the data from LSF stimuli [*F*(2, 54) = 2.89, *p* = 0.044].

There was also a significant interaction between repetition and viewpoint [*F*(2, 72) = 7.23, *p* = 0.0014]. This is an indication that the viewpoint difference between C1 and C2 seems to be a factor in RB for some SF contents.

**Figure 5:**
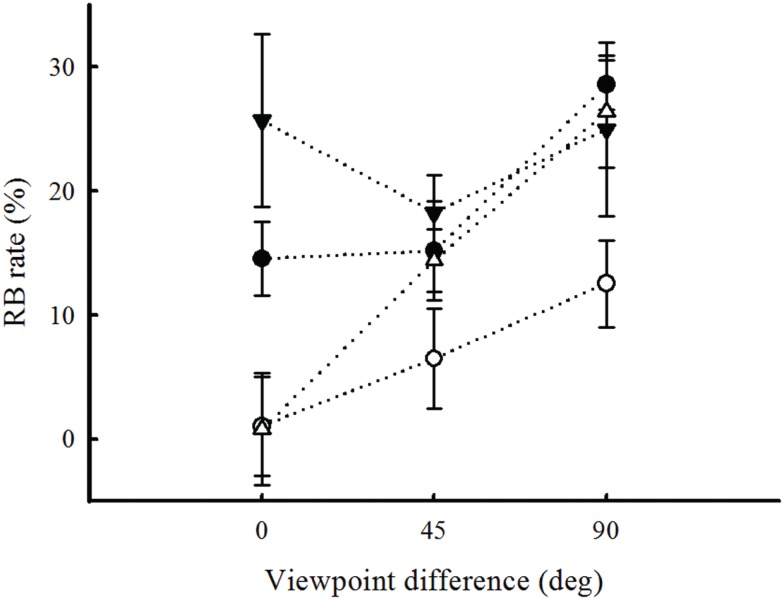
**Repetition blindness rates are plotted as a function of viewpoint for each spatial frequency condition**. RB is the difference between the mean correct-response rate in the non-repeated condition and the mean correct-response rate in the repeated condition. Error bars show ±1 SEM.

The three-way interaction was also significant [*F*(6, 72) = 2.27, *p* = 0.046]. This interaction is also found when we do not take into account the results for the LSF stimuli [*F*(4, 54) = 2.763, *p* = 0.036]. Therefore, additional analyses were performed to determine how RB rates depend on viewpoint for each SF condition separately (see Figure 5). The results revealed that RB was larger when viewpoint differences increased for FS [*F*(2, 18) = 5.3, *p* = 0.015] and HSF images [*F*(2, 18) = 5.36, *p* = 0.014]. In these cases, the highest RB was found for viewpoint differences of 90°. No significant effect of viewpoint on RB was found for the MSF [*F*(2, 18) = 1.5, *p* = 0.24] and LSF conditions [*F*(2, 18) = 0.58, *p* = 0.56]. These results seem counter-intuitive, as one would expect any repetition effect, such as RB, to decrease as images of an object become more different. We come back to this observation in the discussion, in the light of the theoretical framework of RB and object recognition models.

Overall, RB was observed for all kinds of natural and filtered stimuli. Although independent object recognition is viewpoint-invariant, RB was found to increase when the viewpoint difference between the first and second occurrences of the same object was maximized. This indicates that the mechanism leading to RB is viewpoint-dependent.

### Signal-detection analysis

#### *A*′ analysis

On average, *A*′ was equal to 0.83 (±0.12) for the repeated condition, and to 0.895 (±0.09) for the non-repeated condition. The difference was statistically significant [*F*(1, 72) = 386.08, *p* < 0.0001], indicating that C2 was more difficult to discriminate from C1 when C1 and C2 corresponded to the same object (repeated condition) than when they corresponded to different objects (non-repeated condition). Therefore, the *A*′ analysis confirms the existence of an RB effect.

#### *B*″ analysis

*B*″ provides a measure of criterion placement. The difference between *B*″ in the repeated and in the non-repeated conditions gives an idea of the consistency of the observers’ strategy between conditions. Consistent with earlier findings (Arnell and Jolicoeur, 1997, third experiment), we found a more “lax” criterion for the repeated condition (mean *B*″ = 0.29 ± 0.48) than for the non-repeated condition (mean *B*″ = 0.447, ±0.52). The difference was statistically significant between the two conditions [*F*(1, 72) = 49.83, *p* < 0.0001]. Arnell and Jolicoeur (1997) explained their results in terms of the overall difficulty of their task. The relatively low percentages of correct-responses measured in the different conditions of the current study suggest that the task used here was equally difficult to that used by Arnell and Jolicoeur. It is worth noting that observers differed substantially from each other in their respective criterion placement. However, further analysis of the errors showed that the modified criterion did not depend on specific C1–C2 combination.

### Independence analysis

As explained in the material and methods section, this analysis provides a global control. It compares the influence of C1 on C2 in non-repeated situations with correct-response rates in repeated conditions. The significant difference between the measured and the estimated proportion correct [*t* (62) = 9.41, *p* < 0.0001] means that C1 and C2 are not processed independently by the visual system. It provides further evidence that the RB effect depends on the processing of C1. This is in accordance with the model for the RB that relies on the type/token hypothesis. RB is due to the perception of the second occurrence of an object (C2) as a continuation of the first occurrence of the same object (C1). This phenomenon is a failure of the tokenization process, which refers to the attribution of a dedicated occurrence flag for each stimulus.

## Discussion

In this study, we show that RB increases with increasing changes in viewpoint. Moreover, this effect depends on the SF content of the stimulus. Below, we discuss possible explanations for the interaction of viewpoint and SF content in the RB effect.

Traditionally, RB has been interpreted within the theoretical framework of type and token (Kanwisher et al., 1999). In this framework, RB is thought to occur when two occurrences of a same object result in the creation of a single episodic trace (token) for the same object representation (type). Therefore, RB is expected to decrease as the two items become less similar to each other. However, it is important to consider whether similarity refers to physical or semantic properties. In the former case, two physically different views of the same object should lead to decreased RB rate. When objects are rotated in depth, their retinal projections progressively become less similar as the difference in rotation angles (and therefore, the difference in viewpoints) increases, and RB should decrease – even though the objects retain their identity. Although the previous RSVP study of Kanwisher et al. (1999) involving object pictures confirm this prediction, it is worth noting that the viewpoint changes that were used in this study conflated rotation in depth and rotation in the plane, and they included non-canonical views, which could hamper object recognition. Using objects rotated in the picture plane, Harris and Dux (2005) reported that RB disappeared only for upside-down rotations, but remained high for other angles. In contrast, in the present study, which used rotation in depth, RB rate was found to increase with viewpoint changes, at least for FS and HSF pictures. A similar effect of viewpoint was reported by Hayward et al. (2010), although it is worth noting that these authors used line drawings and unfiltered gray-scale pictures.

The fact that the RB rate increased as viewpoint differences increased – and therefore, as physical similarity decreased – suggests that RB cannot solely be accounted for by this physical similarity as already proposed in previous studies (e.g., Kanwisher et al., 1999). It also indicates that even if RB depends entirely on view-based mechanism, that can account for a high-level of tolerance to changes in viewpoint (Dicarlo et al., 2012), the experimental conditions challenge this tolerance, either by the high-level of viewpoint change (up to 90°), or by the temporal constraints of the RSVP stream.

A tentative explanation of the increase in RB rate as a function of viewpoint changes for FS and HSF stimuli is that when an object representation is activated by an oriented template, neighboring templates at different orientations are subsequently activated, as described in several models of object recognition (Tarr and Pinker, 1989; Edelman and Bülthoff, 1992). These models suggest that a part of the representational neural network of an object is activated by the specific viewpoint it encodes. In these models, neural activity spreads over time from the current representation toward other viewpoints that belong to the representational network of the whole object, but with a given delay due to neuronal propagation. The key feature of this proposal is the association between a delayed activation of the neighboring templates, and a decaying activation of the initial template. Assuming these models are plausible biological implementations of object encoding, we speculate that in an RSVP sequence, the feedforward activity elicited by the second occurrence of an object and the lateral spread from a previous viewpoint temporally coincide, such that the two signals merge into a single neural event. That a single neural event is elicited by two successive occurrences of the same – albeit rotated – object could explain why an RB effect was observed for FS and HSF stimuli in the current study.

If this interpretation was correct, the RB rate should follow a U-shaped curve as a function of the delay between the two occurrences of the same object. However, the results obtained with MSF stimuli did not agree with this prediction: RB rate was found to be independent of the viewpoint difference. This result is difficult to explain in the context of the framework described above, and it calls for additional considerations, which are not taken into account in the existing framework. In particular, it is worth noting that MSF stimuli cover a wide range of SFs (range 6 c/deg ± 0.75 octave). Such stimuli with widely spread SFs should elicit responses, the latencies of which are also widely spread in time; for comparison, recall that HSF stimuli are centered on 24 c/deg, with SFs confined to the high-frequency range of the contrast sensitivity function (Legge, 1978). With MSF stimuli that cover a substantial fraction of the SF range, to which the visual system is mostly sensitive, the temporal spreading of the responses to different SFs would lead to blur neural responses to the two occurrences of the same object – in such a way that the whole representation should be activated, and that the RB rate should be independent of the viewpoint. In a functional magnetic resonance imagine (fMRI) study with filtered stimuli representing faces and objects, Goffaux et al. (2011) showed that the brain regions responsible for high-level face representations in humans rely on different SFs over time. For these regions, larger BOLD responses were observed for LSFs than for HSFs for short exposure durations (75 ms), while the reverse pattern of activation was observed for longer durations of exposure (150 and 300 ms). In addition, in the lateral occipital cortex (LOC), BOLD responses to LSFs were never found to be larger than BOLD responses to HSFs, regardless of exposure duration (75, 150, and 300 ms). However, a marked advantage for MSF processing was observed for all exposure durations. The authors of the study concluded that there is no coarse-to-fine processing in the LOC, and that large responses to MSFs are a general feature of high-level visual object processing. In addition, contrast and response latencies are tightly linked to each other (e.g., Gawne et al., 1996). Since in the present study the contrast of the presented stimuli was not homogeneous, this may have caused a larger temporal spreading of the neural responses and to influence the RB effect. To test this prediction, it may be necessary to use stimuli that span a narrower range of SFs and contrasts, so as to reduce as much as possible the variance of the response-latency distribution, and to explore a wide range of delays between the first and second occurrences of the same object. Under such conditions, one expects that RB would be viewpoint dependent for all SFs, and that the highest RB rate would be observed for different delays between the two object presentations.

Another explanation lies in the notion of RB-relief presented by Hayward et al. (2010). They propose to explain a reduced RB for similar viewpoints, instead of a greater RB for large viewpoint differences, in the framework of two competing routes for object recognition: a fast and largely viewpoint-invariant route that relies on local features (Ullman and Bart, 2004), and a whole object route, that is a viewpoint based representation (Hummel and Biederman, 1992; Tarr, 1995). According to Hayward et al. (2010), the lack of a reduction in RB when viewpoints are the same for the two critical items in RSVP (that is, the invariance of RB) is likely due a lesser influence of the whole object route to recognition. Stimuli with MSFs, often related with the diagnostic features that characterize the local feature route, would preferably be encoded via this local feature route, explaining the viewpoint tolerance of RB in this case. In effect, the main difference between MSF and FS would be that in the former case, the object would be encoded as a prototype, whereas in the latter, the object would be encoded as specific instances of an object class. In our experiment, MSF would compare with line drawings and FS with shaded objects. As for HSF, due to the fact that they support mainly detailed information, they would also lead to the encoding as a specific exemplar of an object. Finally, the results obtained with LSF would account for their moderate role in object recognition in our stimuli range. This can be mainly attributed to a relative increased difficulty in figure-ground segregation (Wichmann et al., 2010).

To summarize, we have shown viewpoint-dependency for RB with FS images of natural objects, as well as with HSF-filtered images, but not for MSF filtered images. This suggests that object recognition in RSVP could challenge viewpoint tolerance between view-based templates for FS and HSF images. This viewpoint effect may also be related to the paths *en route* to the activation of the representation, either depending on its sensitization, or on the type of information processed (RB-relief effect).

## Conflict of Interest Statement

The authors declare that the research was conducted in the absence of any commercial or financial relationships that could be construed as a potential conflict of interest.

## Figures and Tables

**Figure A1:**
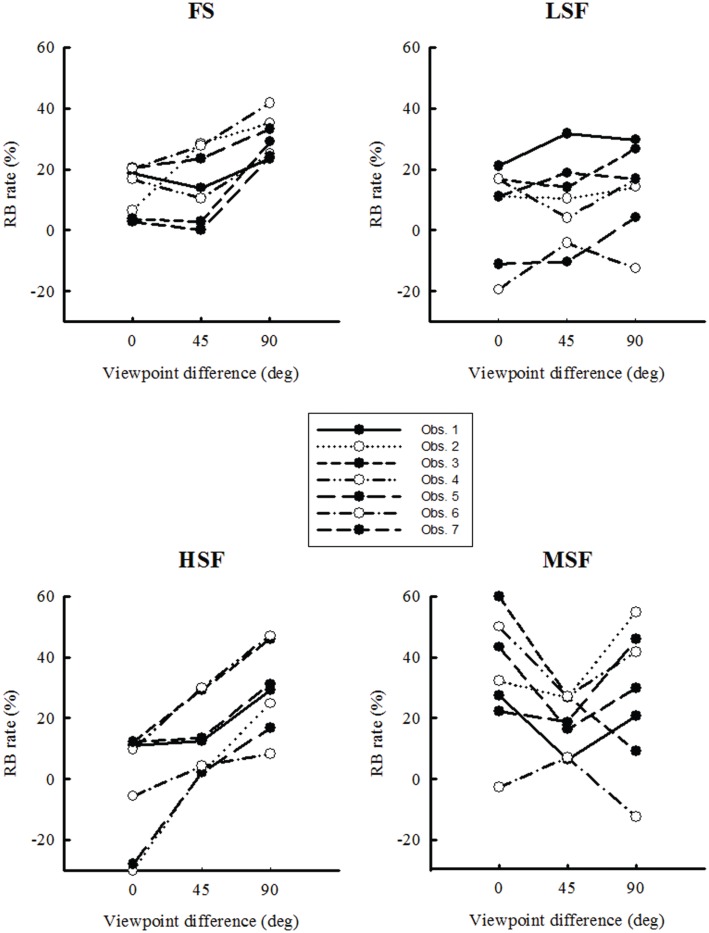
**Repetition blindness rates for each observer are plotted as a function of viewpoint for each spatial frequency condition**. RB is the difference between the mean correct-response rate in the non-repeated condition and the mean correct-response rate in the repeated condition. Error bars are not shown in this figure, to avoid crowding.

**Figure A2:**
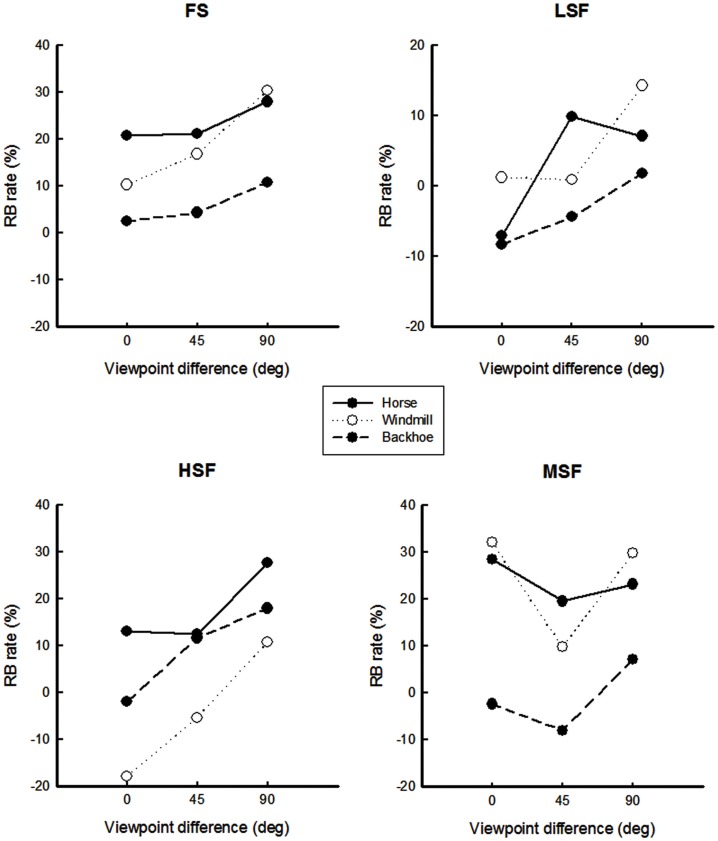
**Repetition blindness rates for each object are plotted as a function of viewpoint for each spatial frequency condition**. RB is the difference between the mean correct-response rate in the non-repeated condition and the mean correct-response rate in the repeated condition. Error bars are not shown in this figure, to avoid crowding.

## Appendix

Here are the detailed computations of the non-parametrical *A*′ and *B*″, derived from Snodgrass and Corwin (1988). We used non-parametric computations, which are more conservative than their parametric counterparts (Arnell and Jolicoeur, 1997).

1. *A*′ is the discrimination index. It estimates the receiving operating characteristic (ROC) area. There are two formulas for the computation of *A*′, given the respective values of Hi and Fa: If Hi ≥ Fa, then
  $$A^{\prime }=\frac{1}{2}+\frac{\left(\text{Hi}-\text{Fa}\right)\left(1+\text{Hi}-\text{Fa}\right)}{4\text{Hi}\left(1-\text{Fa}\right)}$$
  If Hi < Fa, then
  $$A^{\prime }=\frac{1}{2}-\frac{\left(\text{Fa}-\text{Hi}\right)\left(1+\text{Fa}-\text{Hi}\right)}{4\text{Fa}\left(1-\text{Hi}\right)}$$
2. The index *B*″ corresponds to the decision criterion. The computation of *B*″ requires the same precautionary measures as for *A*′: If Hi ≥ Fa, then
  $$B^{\prime \prime }=\frac{\text{Hi}(\text{1}-\text{Hi})-\text{Fa(1}-\text{Fa)}}{\text{Hi}(\text{1}-\text{Hi})+\text{Fa(1}-\text{Fa)}}$$
  If Hi < Fa, then
  $$B^{\prime \prime }=\frac{\text{Fa}\left(1-\text{Fa}\right)-\text{Hi}\left(1-\text{Hi}\right)}{\text{Hi}\left(1-\text{Hi}\right)+\text{Fa}\left(1-\text{Fa}\right)}$$
